# Revision arthroplasty after unicompartimental knee arthroplasty

**DOI:** 10.1186/s13018-021-02767-x

**Published:** 2021-11-12

**Authors:** Nike Walter, Johannes Weber, Maximilian Kerschbaum, Edmund Lau, Steven M. Kurtz, Volker Alt, Markus Rupp

**Affiliations:** 1 Department of Trauma Surgery, University Hospital Regensburg, Franz-Josef-Strauß-Allee 11, 93053 Regensburg, Germany; 2 Exponent Inc, Menlo Park, CA USA; 3 Exponent Inc, Philadelphia, PA USA

**Keywords:** Revision knee arthroplasty, PJI, Unicondylar knee arthroplasty, Exchange arthroplasty, Unicondylar prosthesis

## Abstract

**Background:**

Unicompartimental knee arthroplasty (UKA) is a promising and increasing application to treat unicompartimental knee osteoarthritis. However, revision arthroplasty numbers after UKA are unknown. Therefore, aim of this study was to determine the nationwide burden of revision after UKA by answering the following questions: (1) How did numbers of revision UKA procedures developed over the last decade as a function of age and gender? (2) How high is the percentage of revision UKA procedures due to infection? (3) Which therapy strategy was chosen for surgical treatment of aseptic revision UKA?

**Methods:**

Revision arthroplasty rates as a function of age, gender, infection and type of prosthesis were quantified based on Operation and Procedure Classification System codes using revision knee arthroplasty data from 2008 to 2018, provided by the Federal Statistical Office of Germany (Destatis).

**Results:**

Over the last decade, revision UKA increased by 46.3% up to 3105 procedures in 2018. A trend towards higher numbers in younger patients was observed. Septic interventions constituted 5.7% of all revisions, whereby total procedures increased by 67.1% from 2008 through 2018. The main treatment strategy was an exchange to a bicondylar surface replacement prosthesis, which was done in 63.70% of all cases, followed by exchange to a femoral and tibial shaft-anchored (16.2% of all revisions).

**Conclusion:**

The increasing number of revision arthroplasty after UKA in Germany, especially in younger patients and due to infection, underlines the need for future efforts to improve treatment strategies beyond UKA to delay primary arthroplasty and avoid periprosthetic joint infection.

## Background

Joint replacement is one of the most significant surgical achievement of the twentieth century [1]. The life-enhancing procedure provides pain relief, restores function, and preserves independence, especially in elderly patients. In Germany, primary total knee arthroplasty (TKA) is among the most common surgeries. In 2016, 168,772 TKA procedures were performed. Future numbers of TKA are expected to increase by 45% until 2040 [2]. Especially, unicompartimental knee arthroplasty (UKA) has become popular due to suggested benefits such as less tissue trauma, reduced blood loss during surgery, faster rehabilitation, and improved range of motion [3]. In Germany, UKA procedures nearly tripled over the last decade up to 21,072 performed procedures in 2018 [4]. However, despite good clinical outcomes implant survival is inferior to that of total knee arthroplasties with higher risk for aseptic loosening and periprosthetic fractures [5–7]. Hence, also taken the demographic changes in the industrialized nations into account, increasing number of revision surgeries can be expected. For the U.S. an increase in revision TKA is predicted between 78 and 182% within the next 10 years [8]. However, large registry data on revision UKA is scarce, which makes it difficult to estimate future demands and foresee developments which could be influenced by adaption of prevention and therapeutic measures.

We have therefore aimed to answer the following questions for the Germany population: 1) How did numbers of revision UKA procedures developed over the last decade as a function of age and gender? (2) How high is the percentage of revision UKA procedures due to infection? (3) Which therapy strategy was chosen for surgical treatment of aseptic revision UKA?

## Material and methods

Revision knee arthroplasty data from 2008 to 2018 was provided by the Federal Statistical Office of Germany (Destatis) consisting of annual surgical procedures performed in medical institutions of all 16 German federal states. Since it is mandatory for all somatic German health care providers to settle up costs by the diagnosis related group system, all revision procedures after unicompartimental knee arthroplasty were included to the provided data set. Surgery and procedure keys (Operation and Procedure Classification System codes) were used to identify all unicondylar revision knee arthroplasties in patients aged 20 years or older, regardless of the underlying disease or injury. In particular, the Operation and Procedure Classification System code “5–823.1, exchange of unicondylar prosthesis” was used to retrieve surgical strategies (Table 1). A detailed breakdown of these data by age group and gender was performed. The proportion of prosthetic joint infection was determined by a combination of surgery and procedure keys with the ICD-10 code “T84.5, infection and inflammatory reaction by a joint endoprosthesis” for septic cases and “T84.4, mechanical complication of a joint endoprosthesis” for aseptic cases. Data were analyzed using the software SPSS Version 26.0 (IBM, SPSS Inc. Armonk, NY, USA). Destatis approved the use of data and there is no requirement of consent. Ethical approval was waived by the local institutional ethics committee.

**Table 1:** Operation and procedure classification system code descriptions

Operation and procedure classification system code	Description
5-823.1	Exchange of unicondylar prosthesis
5-823.10 + 5-823.11	Exchange of unicondylar prosthesis to unicondylar prosthesis
5-823.19	Inlay exchange
5-823.1a + 5-823.1b + 5-823.1c	Exchange of unicondylar prosthesis to bicondylar surface replacement prosthesis
5-823.1d + 5-823.1e + 5-823.1f	Exchange of unicondylar prothesis to femoral and tibial shaft-anchored prosthesis
5-823.1x	Exchange of unicondylar prosthesis to other

## Results

**Fig. 1:**
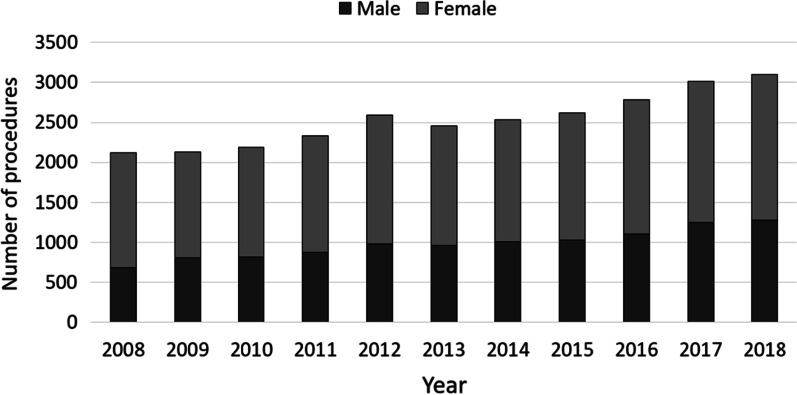
Performed revision procedures after unicondylar knee arthroplasty from 2008 through 2018 divided by gender

**Fig. 2:**
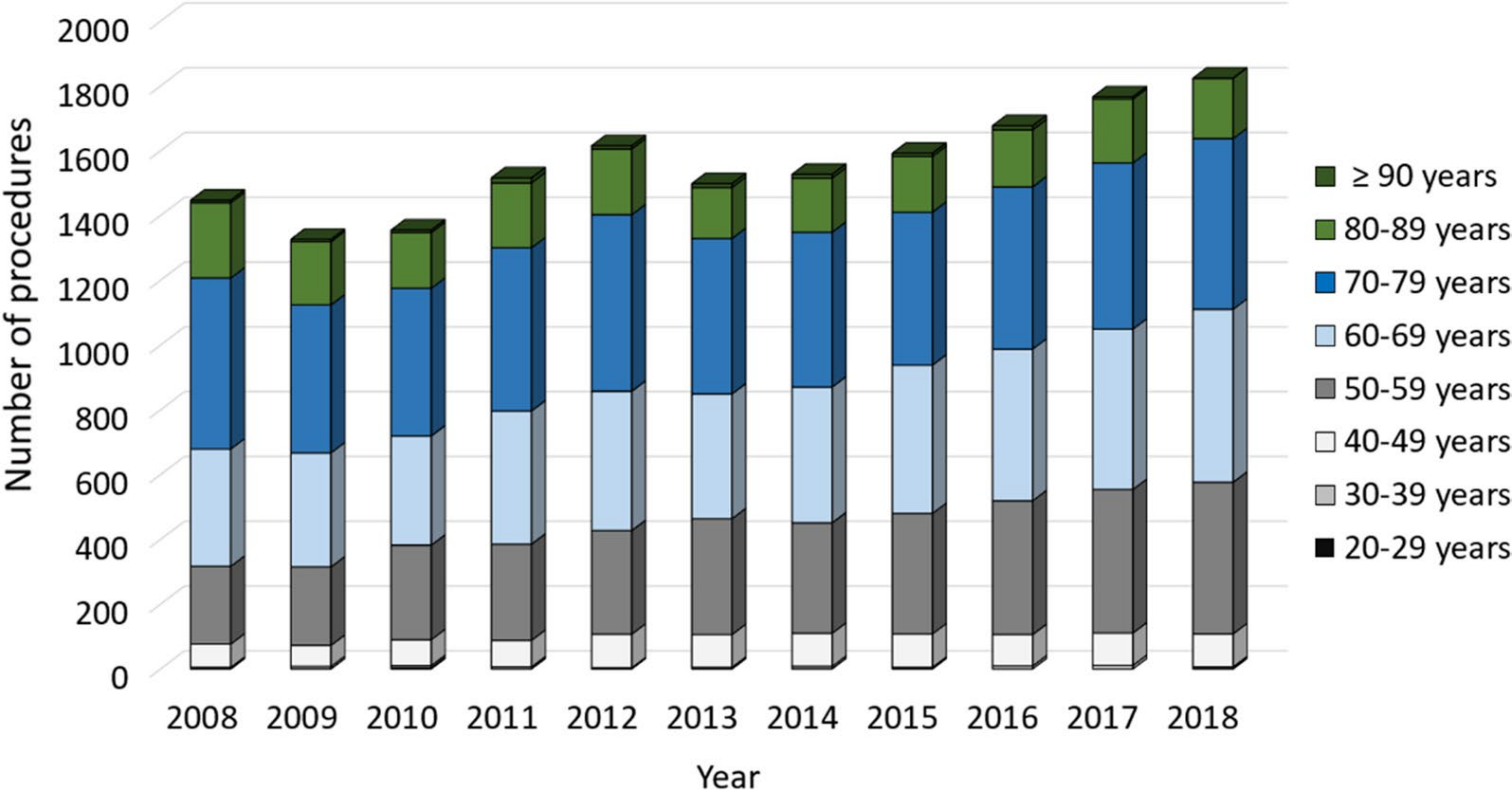
Revision procedures after unicondylar knee arthroplasty of female patients from 2008 through 2018 divided by age

**Fig. 3:**
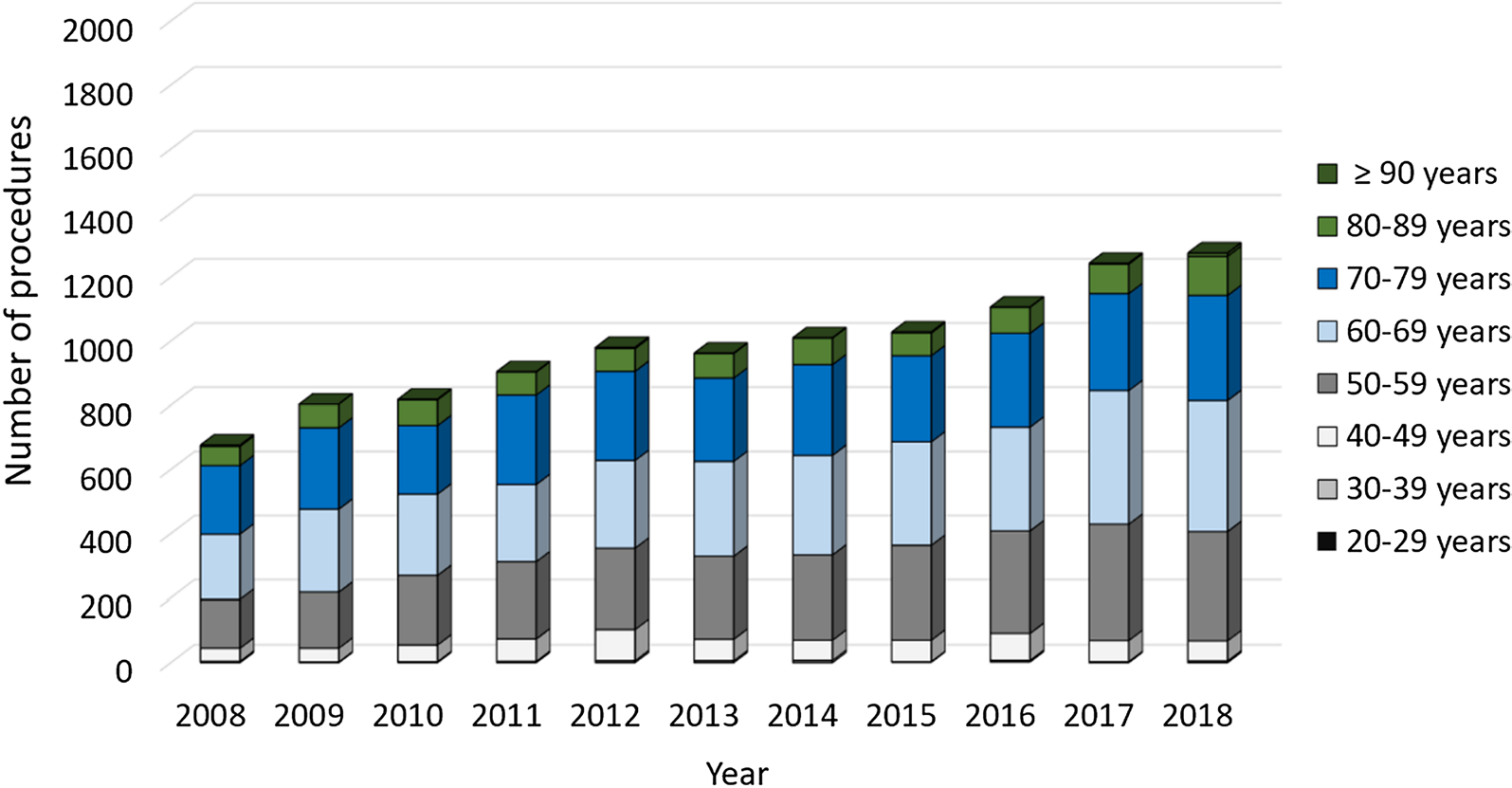
Revision procedures after unicondylar knee arthroplasty of male patients from 2008 through 2018 divided by age

A total of 2123 revisions for unicondylar knee prostheses were performed in 2008. The numbers steadily increased to 3105 procedures in 2018, which represents an overall increase by 46.26% over the last decade (Table 2). Overall, women were more often affected than men (68.2% vs. 31.8%) (Fig. 1). Patients aged 65 years or older comprised the largest cohort with 65.80% of the unicondylar revision cases in 2008, whereas a trend towards revisions in patients younger than 65 years could be observed with an increase of 34.20% to 47.79% between 2008 and 2018. Regarding the female population, unicondylar prostheses revisions performed in patients aged 70–79 years decreased steadily from 36.49% to 28.89% over the last decade, whereas 4.26% more female patients aged 60–69 years underwent a revision surgery in 2018 compared to 2008 and the procedure rates in female patients aged 50–59 years increased from 16.59% to 25.66% (Fig. 2). Similarly, less male patients aged 70–79 years were affected with a decrease from 31.66% in 2008 to 25.73% in 2018, whereas the revision rate for male patients aged 50–59 years and 60–69 years increased by 4.32% and 1.97% over the time, respectively (Fig. 3).

**Table 2:** Development of revision knee arthroplasty after unicondylar knee arthroplasty numbers

Years	Revision unicondylar knee arthroplasty	Relative to 2008 (%)	Septic revisions	Male patients	Female patients	Patients younger than 65 years	Patients aged 65 years or older
2008	2123		70 (3.30%)	676 (31.84%)	1447 (68.16%)	726 (34.20%)	1397 (65.80%)
2009	2129	0.28	91 (4.27%)	803 (37.72%)	1326 (62.28%)	798 (37.48%)	1331 (62.52%)
2010	2188	3.06	85 (3.88%)	818 (37.39%)	1370 (62.61%)	914 (41.77%)	1274 (58.23%)
2011	2329	9.70	113 (4.85%)	873 (37.48%)	1456 (62.52%)	988 (42.42%)	1341 (57.58%)
2012	2595	22.23	124 (4.78%)	980 (37.76%)	1615 (62.24%)	1137 (43.82%)	1458 (56.18%)
2013	2460	15.87	119 (4.84%)	962 (39.11%)	1498 (60.89%)	1153 (46.87%)	1307 (53.13%)
2014	2537	19.50	142 (5.60%)	1010 (39.81%)	1527 (60.19%)	1176 (46.35%)	1361 (53.65%)
2015	2620	23.41	157 (5.99%)	1028 (39.24%)	1592 (60.76%)	1263 (48.21%)	1357 (51.79%)
2016	2783	31.09	160 (5.75%)	1106 (39.74%)	1677 (60.26%)	1374 (49.37%)	1409 (50.63%)
2017	3010	41.78	197 (6.54%)	1244 (41.33%)	1766 (58.67%)	1469 (48.80%)	1541 (51.20%)
2018	3105	46.26	177 (5.70%)	1275 (41.06%)	1830 (58.94%)	1484 (47.79%)	1621 (52.21%)

Septic interventions rates for unicondylar knee prosthesis revisions increased by 39.5% over the time, whereby 70 procedures were performed in 2008 and 177 procedures in 2018 (Table 2). Out of these, 52 surgeries (29.4%) comprised an inlay exchange, 39 procedures (22.0%) were a one-stage exchange and 86 procedures (48.6%) a two-stage exchange. In cases of an exchange, 74.4% recevied a bicondylar surface prosthesis and 25.6% a femoral and tibial shaft-anchored prosthesis.

**Fig. 4:**
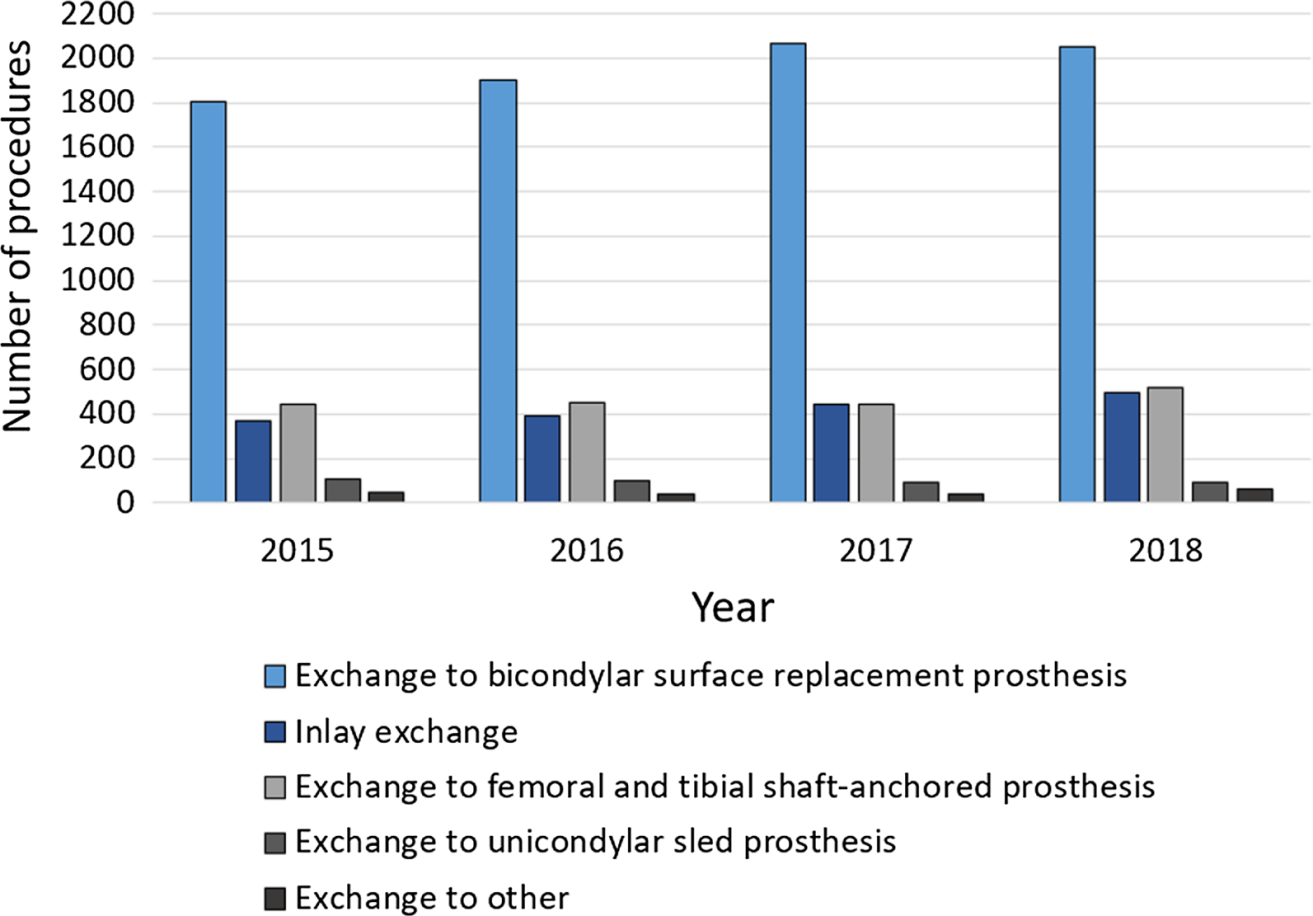
Revision procedures after unicondylar knee arthroplasty from 2015 through 2018 divided by the type of prostheses exchanged to

The main surgical treatment for aseptic unicondylar knee prosthesis revision was an exchange to a bicondylar surface replacement prosthesis, which was done in 63.70% of all cases in 2018. Out of these, 67.31% were conducted in female patients. Absolute numbers increased by 13.51% from 1806 performed procedures in 2015 to 2050 procedures in 2018. The management of choice in 16.19% of all aseptic unicondylar knee prosthesis revisions was an exchange to a femoral and tibial shaft-anchored prosthesis with an increase of performed procedures from 440 in 2015 to 521 in 2018 (15.54%). An inlay exchange was conducted in 15.35% of all cases in 2018, whereby numbers increased by 25.91% from 366 procedures in 2015 to 494 procedures in 2018. Changing an unicondylar knee prosthesis to a new one was done in 2.80% of the evaluated cases, whereas 1.96% underwent an exchange to an unspecified prosthesis (Fig. 4).

## Discussion

Our analysis outlines recent trends in revision UKA from 2008 through 2018 in Germany. Total numbers of revision knee arthroplasty experienced a substantial overall increase during this period (+ 46.3%). Those numbers seem reasonable since advantages in UKA such as less invasive surgery, shorter operative time and hospital stay, lower intraoperative blood loss, higher postoperative range of motion and level of activity, led to an considerable increase in primary UKA numbers [9]. Consecutive higher revision rates, especially when more than half of UKA revisions becoming necessary within five years, can be considered causal for the observed increase in revision UKA [10]. Survivorship after UKA has been reported to be 80.9% and 74.4% in a Medicare and MarketScan cohort and 95.7% and 91.9% for TKA seven years post-surgery. In line with this finding, less frequent survivorship after UKA compared to survivorship of TKA can be accounted for higher revision rates after UKA [11].

Interestingly, our analysis revealed substantially lower revision rates due to PJI after UKA compared to revision due to PJI after TKA, which has been reported to be around 20–30% [12–15]. Reason for lower PJI revision rates in UKA might be the key drivers for aseptic revision surgery after UKA which have been described as osteoarthritis of another compartment and aseptic loosening after UKA [10]. However, as the analysis is based on registry data, the data has to be interpreted with caution. Especially, the underlying cause for revision has to be questioned, as it is possible that infections were not detected in cases coded as aseptic revision.

Depending on remaining bone stock and ligament integrity, revision arthroplasty after UKA can be demanding for the orthopedic surgeon. Although studies dealing with revision UKAs demonstrated less favorable outcome comparable to revision TKA, rates of possible revision types used for conversion from UKA to TKA have not been elucidated, yet [16, 17]. Additional surgical procedure codes for detailed description of revision TKA after unicondylar TKA revealed that most of the performed surgical revisions after unicondylar procedures resulted in conversion to a bicondylar surface replacement arthroplasty. 68.6% (2050) of UKA revisions ended in a bicondylar TKA. Inlay exchange and revision arthroplasty utilizing a shaft-anchored prosthesis are by far less frequently used UKA revision procedures while exchange of UKA to another UKA plays no role in surgical care. No significant change in numbers could be observed for the different subtypes of UKA revision procedures from 2015 through 2018.

### Limitations

The study has several limitations. Historical inpatient data provided by Destatis have been analyzed based on OPS codes, which only allow distinction between different surgical procedures. Inherent limitation of all such analysis is the unverifiable accuracy of coding and data input. Since DRG lump sum reimbursement relies on accurate coding and reimbursement is strictly controlled by the Medical Service of Health Funds, correct coding of diagnosis and procedures can be assumed, however. In addition, reasons for revision UKA could not be itemized beyond revision due to infection using the unspecific ICD code T84.5 (revision arthroplasty due to infection). Since revision UKA is generally performed as an inpatient procedure being reported to the German federal statistical office (Destatis), it can be assumed that the analyzed data set comprises all revision UKA patients in the set time frame. Although, the investigated revision UKA sample can be regarded as complete data set, patient characteristics additionally to gender and age, in general, have not been reported to Destatis, which unfortunately did not allow to analyze driving factors such as comorbidities. Additionally, no information about hospitals and their volume of revision UKA was available which would be of interest in investigating application of treatment strategies and resource utilization.

## Conclusion

The present data demonstrates increasing need for revision surgery after UKA. This increase in revision arthroplasty especially in younger patients and due to infection underlines the need for future efforts to improve treatment strategies beyond partial joint replacement surgery to at least further delay primary arthroplasty and avoid periprosthetic joint infection.

## Data Availability

The datasets used and/or analysed during the current study are available from the corresponding author on reasonable request.
